# Retraction Note: Baicalein mediates inhibition of migration and invasiveness of skin carcinoma through Ezrin in A431 cells

**DOI:** 10.1186/s12885-022-09368-9

**Published:** 2022-03-15

**Authors:** Bin Wu, Ji Li, Damao Huang, Weiwei Wang, Yu Chen, Youxiang Liao, Xiaowei Tang, Hongfu Xie, Faqing Tang

**Affiliations:** 1 Department of Dermatology, Xiangnan College, Chenzhou, 423000 P.R. China; 2 Department of Dermatology, Xiangya Hospital, Central South University, Changsha, 410008 P.R. China; 3 Department of Clinical Laboratory, Xiangya Hospital, Central South University, Changsha, 410008 P.R. China; 4 Metallurgical Science and Engineering, Central South University, Changsha, 410008 P.R. China; 5 Department of Clinical Laboratory, Zhuhai Hospital, Jinan University, Zhuhai, 519000 P.R. China

**Retraction Note: BMC Cancer 11, 527 (2011)**

**https://doi.org/10.1186/1471-2407-11-527**

The Editors have retracted this Article. After publication, concerns were raised regarding the data presented in Figs. 2, 3 and 5. Specifically:

- Fig. 2a and b appear highly similar to Fig. 6a in [1] and Fig. 4c in [2].
- Fig. 3a appears to contain straight line breaks and duplicated patterns in the background of the gel.
- Fig. 5a appears highly similar to Fig. 5a of [1] representing different cell lines.

Th﻿e authors provided the raw data to address these concerns, but the images contained further overlap with [1]. The Editors therefore no longer have confidence in the presented data.

Author Faqing Tang has not specifically stated whether they agree with the retraction. Authors Weiwei Wang, Yu Chen and Hongfu Xie have not responded to any correspondence from the Editor or Publisher about this retraction. The Publisher has not been able to obtain current email addresses for authors Bin Wu, Ji Li and Damao Huang.

[1] Huang, D., Wang, W., *et al*. Berberine inhibits the invasion and metastasis of nasopharyngeal carcinoma cells through Ezrin phosphorylation. *J. Cent. South Univ. (Med. Sci.)* 36,7, 616-23 (2011). 10.3969/j.issn.1672-7347.2011.07.006

[2] Tang, F., Wang, D., *et al*. Berberine inhibits metastasis of nasopharyngeal carcinoma 5-8F cells by targeting Rho kinase-mediated Ezrin phosphorylation at threonine 567. *J. Biol. Chem.*, 284(40), 27456-27466 (2009). 10.1074/jbc.M109.033795

